# The Qatar 2022 World Cup warm-up: Football goal-scoring evolution in the last 14 FIFA World Cups (1966–2018)

**DOI:** 10.3389/fpsyg.2022.954876

**Published:** 2023-01-04

**Authors:** Branimir Mićović, Bojan Leontijević, Milivoj Dopsaj, Aleksandar Janković, Zoran Milanović, Amador Garcia Ramos

**Affiliations:** ^1^Department of Sports Games, Faculty of Sport and Physical Education, University of Belgrade, Belgrade, Serbia; ^2^Department of Motoric and Methodology, Faculty of Sport and Physical Education, University of Belgrade, Belgrade, Serbia; ^3^Department of Physical Education and Health, Institute of Sport, Tourism and Service, South Ural State University, Chelyabinsk, Russia; ^4^Faculty of Sport and Physical Education, University of Niš, Niš, Serbia; ^5^Science and Research Centre Koper, Institute for Kinesiology Research, Koper, Slovenia; ^6^Incubator of Kinanthropology Research, Faculty of Sports Studies, Masaryk University, Brno, Czechia; ^7^Department of Physical Education and Sport, Faculty of Sport Sciences, University of Granada, Granada, Spain; ^8^Department of Sports Sciences and Physical Conditioning, Faculty of Education, Universidad Catolica de la Santísima Concepcion, Concepción, Chile

**Keywords:** football (soccer), goal scoring, World Cup, tactics, performance analysis

## Abstract

The aim of this study was to elucidate pattern of attacking actions leading up to goal scoring during the 14 FIFA World Cups from 1966 to 2018. The study analysed 1881 goals scored during a total of 732 matches. We employed observational methodology design. Before goal analysis began, it was developed the observing protocol in which data related to selected variables, by system of notation, was entered after reviewing each individual goal scoring action. The analysis of all video material was carried out independently by four experienced examiners (three of them are Ph.D in sports science and one is Ph.D. candidate in sports science with at least 7 years of coaching and experience as analyst in football). The inter-and intra-observer reliability presented good level of agreement. The kappa values ranged from 0.82 (goal scoring through open play) to 1.00 (action leading up to goal), showing a very high agreement for all performance variables. Interclass correlation was very high (ICC = 0.966, 95% upper and lower confidence intervals were between 0.933 and 1.00). A statistically significant trend (*p* < 0.05) from 1966 to 2018 was identified towards a higher relative frequency of goals scored from set play and collective actions from open play. The Chi-square did not reveal significant differences in the frequency of goal scoring patterns and goal-scoring zones. The results also revealed that the majority of goals were scored between the 76th and 90th minutes of a match (22.7%), from open play (70.5%), inside the penalty area (54.7%), one touch finishing (62.5%), and collective attacks in open play (55.8%). These findings may provide a possible strategic direction for improving goal-scoring performance in football, as well as practical implementation in World Cup tournament preparation.

## Introduction

In football, the team’s performance is affected by a complex interaction of technical, tactical, physical, and mental factors (Drust et al., 2007). Due to this complexity, by only watching live matches, coaches cannot accurately collect, analyse, understand and interpret the necessary information to draw proper conclusions about the football game (Franks and Miller, 1986). Therefore, it is necessary to examine competitive actions in to collect meaningful data on the performance of football teams and individual players. For this purpose, the observational methodology is used to collect information in team sports, as it allows for the collection of multiple variables that interact in the sporting context (Anguera, 2003; Anguera and Hernandez-Mendo, 2015). The use of observational methodology in the sports context provides coaches and other sports professionals with flexible tools that adapt to their needs (Ortega-Toro et al., 2019).

The most important factor that determines the result in football is goal (Carling et al., 2005; Hughes and Franks, 2005; Cachay and Thiel, 2000; Cebi et al., 2016). Goal scoring, as a key factor in a team’s success, is one of the variables more investigated during match analyses (Yiannakos and Armatas, 2004; Armatas and Yiannakos, 2010; Lago-Peñas et al., 2011; Muhammd et al., 2013; Wallace and Norton, 2014; Rumpf et al., 2017; Vergonis et al., 2019). However, the majority of previous research has been limited to a single championship, either at the national or international level (Durlik and Bieniek, 2014; Janković et al., 2011; Cebi et al., 2016; Vergonis et al., 2019; Kubayi, 2020). However, there is little data regarding how the football play has evolved over multiple decades.

The overall tactics of football entail a permanent interrelationship between the patterns of attacking and defensive play (Barreira et al., 2014). The evolution of football has resulted in changes in the physical, tactical, and technical requirements of the game. In a tactical sense, the changes mostly refer to a reduction in the playing area (Wallace and Norton, 2014). By examining the final matches of FIFA World Cups from 1966 to 2010, it was noted that the movements of the ball and, therefore, the speed of play increased by 15% (Wallace and Norton, 2014). A major change has been observed in the number of passes, which increased from 10.75 passes/min in 1966 to 14.71 passes/min in 2010 (35% increase). Moreover, ball recovery form represents important patterns of attacking play in the World Cup semi-final stage (Barreira et al., 2014). All of these physical, tactical, and technical changes have shaped football into more dynamic game. The question is whether and how all these changes have affected goal scoring actions in recent decades, specifically goal scoring at World Cups as a key indicator of football development.

By an overview of the latest research, it may be concluded what a goal scoring action looks like in various professional tournaments. In the 2016/17 UEFA Champions League season, 75.9% of goals were scored from open play (González-Ródenas et al., 2021). Most of the previous studies have shown that between 20 and 30% of goals were scored from set play (Yiannakos and Armatas, 2004; Armatas and Yiannakos, 2010; Njororai, 2013; Gonzalez-Rodenas et al., 2019). In addition, 63.9% of goals started from the offensive third of the pitch, 23.1% from the middle third of the pitch, and 13.0% from the defensive third of the pitch (Vergonis et al., 2019). Moreover, 50.5% of goals were scored with less than two passes performed in the action, 19% of goals with 3 to 4 passes, and 31.1% goals with more than 4 passes (Kubayi, 2020). Short passing (<30 metres) were performed in 69.9% of the goal scoring actions (Kubayi, 2020). Collective attacking actions represented 51.6% of the goal scoring actions, whereas 10.5% of the actions leading up to goals were achieved through individual attacks 60.3% of the goals from open play were scored with one touch (González-Ródenas et al., 2021).

Only few studies have examined the goals scored in multiple consecutive World Cups. Leite (2013) analysed the goal scoring periods in the FIFA World Cups from 1930 to 2010 whilst Armatas et al. (2007) examining three consecutive World Cups (1998, 2002 and 2006) and both studies concluded that most goals were scored in the period from the 76th minute until the end of the match. However, aforementioned authors did not provide a complete movement pattern ahead of goal scoring, therefore it is hard to identify key technical and tactical elements from their studies. In contrast, Kubayi and Toriola (2019) analysed the ways in which goals were scored in five consecutive World Cups in the period between 1998 and 2014 and found that most goals were scored from inside the goal area (23.8%) and penalty area (53.6%) in addition to the results regarding the goal scoring periods (most goals scored in the period from the 76th minute to the end of the match, i.e., 24.7%). This study provided a more complete picture regarding how goals were scored over a 20-year period. Generally there is a lack of analysis over a longer period to draw parallels between different periods of football development and stay still unknow how these specific elements related to goal scoring situations have changed through the history of football.

Therefore, the aim of this study was to elucidate pattern of attacking actions leading up to goal scoring during the 14 FIFA World Cups from 1966 to 2018 including an analysis of the moment of the goal, the technical action and some tactical aspects prior to the goal being scored. We hypothesised that game period and goal scoring zones as well as players’ individual characteristics will change constant throughout football World Cups history.

## Materials and methods

### Sample of matches

732 matches played during 14 consecutive FIFA World Cups in the period between 1966 and 2018 were analysed. In that period, there were a total of 1881 goals scored in FIFA World Cups, which represented the sample analysed in this study (the goals scored in penalty shootouts were not taken into account). The research was conducted according to the postulate of the declaration of Helsinki and with permission of the Ethics Committee of the University of Belgrade Faculty of Sport and Physical Education (02 No. 484–2).

### Data collection procedures

The video material for the analysis was obtained from the website https://footballia.net, which contains videos of all of the previous 14 FIFA World Cup matches. Each goal scoring action was captured in a video clip using the iMovie software. The data for goal scoring timing were obtained from the FIFA website, https://www.fifa.com. Before goal analysis began, it was developed the observing protocol (Carling, 2008), in which data related to selected variables, by system of notation, was entered after reviewing each individual goal scoring action (O’Donoghue, 2010). The analysis of all video material was carried out independently by four experienced examiners (three of them are Ph.D in sports science and one is Ph.D. candidate in sports science with at least 7 years of coaching and experience as analyst in football). The operational definitions of the variables considered in this study are displayed in Table 1, and the ICC values for each of the variables are included.

**Table 1:** Operational definition of the dependent variables analysed in the present study.

Variable	Description	Crombah’s alpha	ICC (95% confidence interval)	*p*
Frequency of goals scored Kubayi and Toriola (2019) and Vergonis et al. (2019)	Per half (first half, second half, extra time), 15 – min period (1–15, 16–30, 31–45+, 46–60,61-75,76–90+), and 15-min period of extra time (91–105+, 106–120+)	0.835	0.795 (0.566–0.924)	0.000
Type of play Vergonis et al. (2019) and Kubayi (2020)	Open play or set play	0.781	0.776 (0.302–0.928)	0.006
Goal scoring zones Njororai (2013) and Cebi et al. (2016)	Penalties, inside the goal area (5 m), inside the penalty area (<16 m) and outside the penalty area (16 m>)	0.777	0.722 (0.372–0.900)	0.001
Actions leading up to goal Gonzalez-Rodenas et al. (2019)	One touch (1), two touches (control + shot, 2) and more than two touches (2+)	0.686	0.523 (−0.170–0.834)	0.053
Body part used to score a goal Njororai (2013) and Durlik and Bieniek (2014)	Right leg (RLEG), left leg (LLEG), head and own goal (OwnG)	0.780	0.689 (0.297–0.888)	0.002
Penultimate action - goals from open play Gonzalez-Rodenas et al. (2019)	Technical – tactical action performed immediately before the final action that allows the final player to have the opportunity of shooting at goal. This action may be performed by the same player that shoots at goal (individual action) or by a teammate that passes the ball to the final player (collective play). Individual action: *Dribbling* (the final player dribbles the ball past defenders to create a goal scoring opportunity), *Running with the ball – Carrying* (the final player carries the ball towards a goal scoring situation), *Collecting a free ball (Free ball)* (the final player collects a free ball that allows him to have an immediate scoring opportunity) *Shot from distance (Shot FD)* (the final player shoots outside the penalty area). Collective play: *Deep-pass* (pass that breaks the opposing defensive line and allows the receiver to have an immediate scoring opportunity in front of the goalkeeper), *Cross* (pass performed from the wide channels of the field in the opposing half towards the penalty box that allows the receiver to have an immediate scoring opportunity), *Back pass* (pass that is performed backward in relation to the player who performs the final pass and that allows the receiver to have an immediate goal scoring opportunity).			
		0.065	−0.110 (−1.506–0.601)	0.560
		0.628	0.604 (0.029–0.862)	0.022

### Reliability testing

An intra-observer test using the Cohen’s kappa measure of agreement was performed to assess the reliability of all variables, and rated using the following criteria: ≤0 as indicating no agreement and 0.01–0.20 as none to slight, 0.21–0.40 as fair, 0.41–0.60 as moderate, 0.61–0.80 as substantial, and 0.81–1.00 as almost perfect agreement (Cohen, 1960). We randomly selected 244 out of the 1881 goals (13%) and the main researcher analysed and re-analysed them 3 weeks later to reduce the learning effect (O’Donoghue, 2010). The kappa values ranged from 0.82 (goal scoring through open play) to 1.00 (action leading up to goal), showing a very high agreement for all performance variables (Kubayi, 2020). We used Interclass Corelation coefficient (ICC) to calculate inter – observer reliability. ICC estimates and their 95% confident intervals were calculated using a mean-rating, absolute-agreement, two-way mixed-effects model (Koo and Li, 2016). Interclass correlation was very high (ICC = 0.966, 95% upper and lower confidence intervals were between 0.933 and 1.00).

### Statistical analysis

All the variables were entered into a Microsoft^®^ Excel^®^ for Mac 2011, Microsoft Corporation, spreadsheet. All data analyses were performed using IBM SPSS Statistics (v19.0; IBM Corp., Armonk, NY, United States). A Chi-square test was used to identify statistically significant differences in goal scoring frequency. For effect size calculating we used Cramer’s V (Akoglu, 2018). Effect sizes (Cramer’s V) were determined for each test and rated using the following criteria: small = 0.10; medium = 0.30; strong/large = 0.50 (Cohen, 1988). A linear regression analysis was used to determine the trend of change in the investigated variables with World Cups serving as the independent variable. Statistical significance was set at *p* ≤ 0.05 (O’Donoghue, 2010).

## Results

There have been 1881 goals scored in the previous 14 FIFA World Cups. The 1970 World Cup had the most goals (an average of 2.97 per match), whilst the 2010 World Cup had the fewest (an average of 2.27 per match). The second-half scoring rate was 56.3%, whilst the first-half and overtime scoring rates were 41.1 and 2.6%, respectively. The majority of goals were scored between the 76th and final minutes of the game (22.7%). However, no significant differences through the examined period (1966–2018) were found for the distribution of goal scoring periods [χ^2^ (65, *n* = 1831) = 81.4, *p* = 0.082, *V* = 0.094] (Table 2).

**Table 2:** Relative distribution (%) of the frequency of goals scored per 15-min period and 15-min period of extra time.

Interval (minutes)	1966	1970	1974	1978	1982	1986	1990	1994	1998	2002	2006	2010	2014	2018	χ^2^	Cramer’sV	*p*
0–15	18.0	12.6	13.4	11.8	11.0	13.6	7.0	15.6	14.0	16.1	15.6	9.7	10.5	11.8	81.4	0.094	0.082
16–30	12.4	14.7	17.5	9.8	15.1	13.6	11.3	14.2	11.7	13.0	16.3	15.9	15.8	10.1			
31–45+	16.9	8.4	15.5	28.4	8.2	13.6	10.4	16.3	15.2	16.1	15.6	15.2	11.7	15.4			
46–60	12.4	21.1	17.5	13.7	19.9	15.9	16.5	16.3	18.1	16.8	12.2	15.2	14.0	21.3			
61–75	14.6	18.9	16.5	18.6	26.7	18.9	20.0	15.6	14.6	18.0	8.8	18.6	19.3	17.8			
76–90+	23.6	16.8	19.6	15.7	17.1	20.5	30.4	19.9	25.7	18.0	29.3	24.1	24.0	21.9			
**Extra time**																	
91–105+	1.1	3.2	0.0	1.0	2.1	1.5	0.9	1.4	0.0	1.2	0.7	0.7	1.8	0.6			
106–120+Games	1.1	4.2	0.0	1.0	0.7	2.3	3.5	0.7	0.6	0.6	1.4	0.7	2.9	1.2			
	32	32	38	38	52	52	52	52	64	64	64	64	64	64			
GPM	2.78	2.97	2.55	2.68	2.81	2.54	2.21	2.71	2.67	2.52	2.3	2.27	2.67	2.64			

Games – Number of games; GPM – Goals Per Match.

The distribution of goal scoring zones did not significant change through the examined period [χ^2^ (39, *n* = 1,881) = 41.6, *p* = 0.359, *V* = 0.086], whilst significant differences were obtained for the distribution of actions leading up to goals [χ^2^ (26, *n* = 1,648) = 40.1, *p* = 0.038, *V* = 0.110] and the body parts used to score the goals [χ^2^ (52, *n* = 1,881) = 78.1, *p* = 0.011, *V* = 0.102] (Table 3). Most goals were scored from inside the penalty area (54.7%), followed by inside the goal area (21.3%), outside the penalty area (15.6%), and finally from the penalty spot (8.9%). One touch finishing was the dominant way of scoring goals (62.5%), followed by two-touches (19.2%), and finally more than two touches (18.3%). Most of the goals were scored with the right leg (51.6%), followed by the left leg (28%), head (17.8%) and own goals (2.6%).

**Table 3:** Relative distribution (%) of goal scoring zones, actions leading up to a goal, and body parts used to score a goal.

		1966	1970	1974	1978	1982	1986	1990	1994	1998	2002	2006	2010	2014	2018	χ^2^	Cramer’sV	*p*
Scoring zones	5 m	24.7	22.1	22.7	12.7	21.9	22.0	19.1	16.3	21.1	21.7	25.2	24.1	24.6	18.3	41.6	0.086	0.359
	<16 m	55.1	51.6	54.6	56.9	52.1	58.3	56.5	51.1	56.1	55.3	49.0	51.7	57.3	54.4			
	16 m>	11.2	21.1	16.5	18.6	20.5	10.6	13.0	22.0	12.3	14.3	17.0	17.9	11.1	14.2			
	Penalty	9.0	5.3	6.2	11.8	5.5	9.1	11.3	10.6	10.5	8.7	8.8	6.2	7.0	13.0			
Action leading up to goal	1	64.6	53.5	69.0	53.5	61.1	66.1	62.9	58.3	54.8	73.4	61.4	62.8	60.6	70.9	40.1	0.110	0.038
	2	16.5	16.3	20.7	26.7	16.8	19.5	19.6	20.0	26.0	13.7	17.3	19.4	23.2	14.9 14.2			
	2+	19.0	30.2	10.3	19.8	22.9	14.4	17.5	21.7	21.2	12.9	21.3	17.8	16.1				
Body part	RLEG	59.6	53.7	49.5	53.9	47.3	52.3	55.7	57.4	52.0	41.6	59.9	57.2	40.9	46.7	78.1	0.102	0.011
	LLEG	22.5	32.6	26.8	30.4	35.6	31.1	19.1	24.8	25.1	33.5	19.0	22.8	36.8	27.2			
	Head	13.5	12.6	20.6	12.7	16.4	14.4	25.2	17.0	18.1	21.1	18.4	17.2	18.7	18.9			
	OwnG	3.4	1.1	3.1	2.9	0.7	1.5	0.0	0.7	3.5	3.1	2.7	2.1	2.9	7.1			

5 m – Inside the goal area; <16 m – Inside the penalty area; 16 m > − Outside the penalty area; 1 – One touch; 2 – Two touches; 2+ − More than two touches; RLEG – Right leg; LLEG – Left leg; OwnG – Own goal.

No significant differences between the examined period were noted for the distribution of different types of final passes performed in collective attacks [χ^2^ (39, *n* = 739) = 37.5, *p* = 0.068, *V* = 0.143] or the distribution of occurrences of individual actions leading up to goal scoring [χ^2^ (39, *n* = 686) = 49.7, *p* = 0.116, *V* = 0.155] (Table 4). According to the data from the final pass analysis, the majority of goals from collective attacking actions (46.6%) were scored following deep passes, followed by crosses (39.1%), and back passes (14.3%). When it comes to individual actions that resulted in goals, 39.7% were scored after collecting a free ball, 33.4% from shots from distance, 19.6% from dribbling, and 7.3% from carrying the ball. There were 70.5% of goals scored from open plays, whilst 29.5% of goals were scored from set plays. In terms of the final actions leading up to goals from open play, 55.8% of goals were scored after a teammate’s pass and 44.2% as a result of individual actions.

**Table 4:** Relative distribution (%) of penultimate actions leading up to goal scoring.

		1966	1970	1974	1978	1982	1986	1990	1994	1998	2002	2006	2010	2014	2018	χ^2^	Cramer’sV	*p*
Collective play	Deep pass	48.3	33.3	31.7	44.7	43.8	55.0	42.9	58.8	59.7	42.6	55.9	40.0	42.3	42.3	37.5	0.143	0.068
	Back pass	4.2	17.9	26.8	15.8	21.1	5.0	11.9	7.8	8.1	9.3	15.3	18.3	20.5	17.3			
	Cross	37.5	48.7	41.5	39.5	35.1	40.0	45.2	33.3	32.3	48.0	28.8	41.7	37.2	40.4			
Individual Action	Dribbling	16.7	35.0	3.1	23.8	13.2	21.6	20.0	20.5	27.5	29.7	14.7	15.2	14.9	16.7	49.7	0.155	0.116
	Carrying	4.8	5.0	12.5	2.4	11.3	0.0	11.4	9.1	11.8	2.7	5.9	4.3	10.6	8.3			
	Free ball	57.1	20.0	43.8	35.7	34.0	48.6	37.1	29.5	33.3	27.0	32.4	34.8	36.2	35.4			
	Shot FD	21.4	40.0	40.6	38.1	41.5	29.7	31.4	40.9	27.5	40.5	47.1	45.7	38.3	39.6			

**Figure 1:**
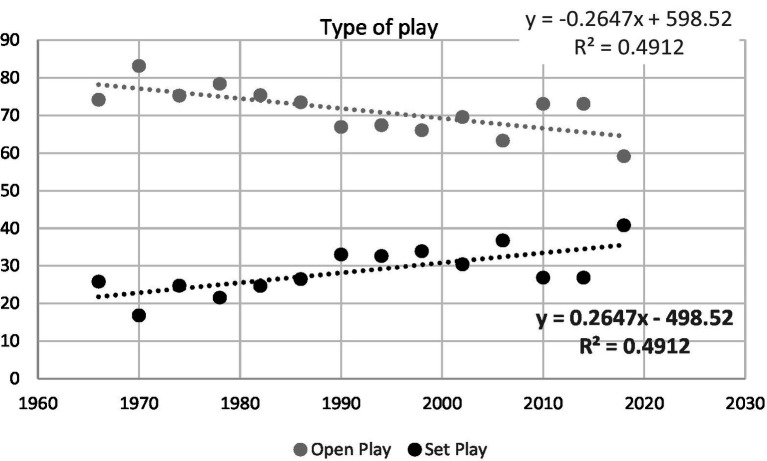
Regression analysis of the frequency of the different types of play (open and set play) through the 14 FIFA World Cups.

**Figure 2:**
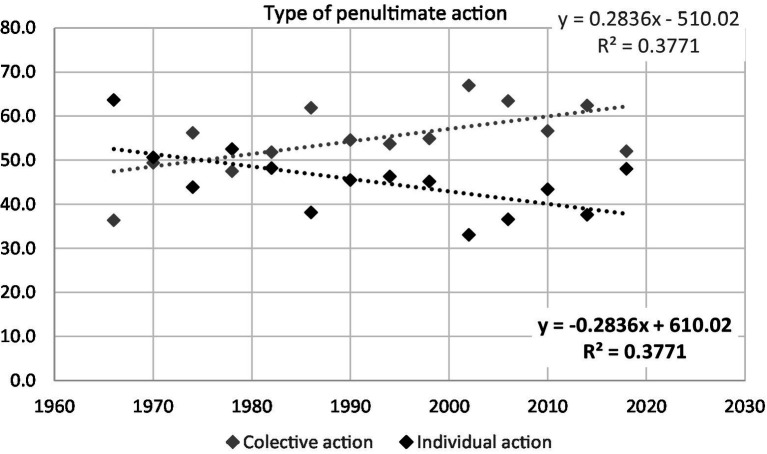
Regression analysis of the frequency of the type of penultimate action (collective and individual actions) through the 14 FIFA World Cups.

Linear regression analyses only showed a statistically significant trend through the succession of 14 FIFA World Cups for the different types of play (open play or set play; *R*^2^ = 0.49, *F* = 11.6, *p* = 0.001; Figure 1) and the penultimate action preceding the goal (collective action or individual action; R^2^ = 0.33, *F* = 7.3, *p* = 0.019; Figure 2). In the period between 1966 and 2018, per each World Cup the frequency of goals scored from the set plays increased by 0.27% and the goals promoted by collective actions increased by 0.28%.

## Discussion

The aim of this study was to elucidate pattern of attacking actions leading up to goal scoring during the 14 FIFA World Cups from 1966 to 2018. In line with previous studies (Armatas et al., 2007; Leite, 2013) here was an upward trend in the number of goals scored as the game progressed. Most goals were scored in the last 15 min of a match. There are same findings found by many previous research of major professional competitions: UEFA Champions League, English Premier League, French Football League 1, Italian Seria A, Spanish football Liga (Alberti et al., 2013; Durlik and Bieniek, 2014; Michailidis et al., 2018; Zhao and Zhang, 2019).

In 11 out of the last 14 World Cups, most goals were scored during the last period of the match. Since 1986, i.e., in each of the 9 previous tournaments, the majority of goals were scored between the 76th minute and the end of the match. These findings indicate that this period of the game is critical for changing the score at World Cups. It is important to note that, since 1990, a greater number of goals have been scored after the 90th minute of a match (overtime). This trend reached its peak in the last two tournaments (2014 and 2018).

A change in scoring in the final 15 min of a match, particularly in the final minutes, could be attributed to several factors. Physical performance has been proven to decline throughout the second half (Carling and Dupont, 2011) whereas mental fatigue increases as the match progresses towards the end (Barte et al., 2017). All of this could result in a higher number of technical errors, which could directly affect the score (Russell et al., 2011). In a tactical sense, teams that want to and strive to score a goal will take more risks and organise attacks with more players, attempting to reach the opponent’s goal as quickly as possible (Abt et al., 2002). Such a style of play can create more opportunities for the opposing team’s counter-attacks and make the attacking team’s defence more vulnerable (Gonzalez-Rodenas et al., 2019). As a result, physical and mental fatigue, combined with changes in technical and tactical aspects, can evidently lead to a higher number of goals in the final minutes of the game.

Since the 1970 World Cup, when most of the goals were scored from open play (83.2%), there has been an upward trend in the number of goals scored from set play. Before 1990 the percentage of goals scored from set play ranged from 21.6 to 26.5%, whilst after 1990 ranged from 30.5 to 36.8% (with exception of WC 2010 and 2014). The significance of set play in World Cup goal scoring reached its peak in 2018, when this type of attack produced 69 goals (40.8%). Those findings coincide with those found in study from Kubayi and Toriola (2019) which analysed the 795 goals scored during a total of 320 matches played in five successive FIFA World Cup tournaments (1998–2014). Results shown that most set plays goals were scored in 1998 (36.3%) and 2006 (31.3%), declining to 24.1% and 22.2% in 2010 and 2014, respectively. Also, finding from other studies, focused on elite, shown that set pieces have been shown to produce approximately 30% of goals in recent international tournaments (Armatas and Yiannakos, 2010; Mitrotasios and Armatas, 2014; Gonzalez-Rodenas et al., 2019).

It is possible that scoring goals from open play is becoming more difficult in the World Cup due to a decrease in the free playing area, an increase in player density in space, and a need to make technical and tactical decisions faster (Pollard et al., 2004; Wallace and Norton, 2014). However, when set play is used, it is easier to shoot the ball because it remains stationary on the ground, opposing players must remain at some distance from the ball, and the attacking team can choose the moment to start the action. Being close to the opponent’s goal enables sending the ball into the penalty box with the immediate intention to score a goal and more players can be positioned in front of the opposing goal (Zileli and Söyler, 2020). All of the aforementioned characteristics could influence training plans to devote more time to set play (both attacking and defending), particularly in the few weeks that national teams have to prepare for a major tournament.

In each of the tournaments analysed, most of the goals were scored inside the penalty area followed by the goal area. Other studies dealing with the zones from which goals are scored in football have confirmed a significantly higher number of goals scored from within the penalty box (inside the penalty area + inside the goal area; Yiannakos and Armatas, 2004; Armatas and Yiannakos, 2010; Janković et al., 2011). This is not surprising since the penalty box is the zone located nearest to the goal within which a goal-scoring probability is much higher compared to zones that are more distant from the goal (Pratas et al., 2018). Another possible explanation for the higher number of goals scored from within the penalty area is the players’ preference to be near the goalpost, but not in the goalkeeper’s range so that they can shoot the ball with less distraction (Muhammd et al., 2013). The fact that fewer goals were scored by shooting from outside the penalty box can be attributed to compact defence structures in this zone as well as to the goal-scoring probability that decreases as the distance between the goal and the location of a player is increased (Pollard et al., 2004). It should be hereby highlighted that there was a record number of goals scored from penalty kicks in the last World Cup (22, i.e., 13.8%). An increased number of goals scored from penalty kicks can also be explained by using the VAR technology in 2018 (Vergonis et al., 2019).

Players rarely have time for extra contact with the ball due to the close proximity of the opposing team’s players and goalkeeper in the zones where most goals are scored (penalty box; González Rodenas et al., 2020). Furthermore, the goalkeeper’s ability to react is hampered by the player’s quick shot (Durlik and Bieniek, 2014). From a technical standpoint, a decent level of technical training is required to shoot the ball with one touch towards or into a desired area because quick reactions and precise shots are required, it is recommended that attackers spend the majority of their training time shooting the ball with one touch (Sarmento et al., 2018).

When it comes to goals scored from open play (Figure 2), the results of this study have indicated that the number of goals scored after individual actions have been decreasing in favour of collective actions. In accordance with our results, previous literature that analysed goal patterns in the Eurocup 2004 (Yiannakos and Armatas, 2004), World Cup 2006 (Armatas and Yiannakos, 2010), English Premier League 2008/2009 (Durlik and Bieniek, 2014) and Eurocup 2012 (Mitrotasios and Armatas, 2014) found that less than 20% of goals were scored after individual actions.

In the evolution of football, the collaboration of two players in the finishing actions of an attack is becoming a more important element. Consequently, the player who is capable of delivering a final pass to his teammate can be considered as important as a goal-scorer (Gonzalez-Rodenas et al., 2019). An increase in the number of goals scored as a result of collective play can be explained by the growing quality of team’s defence (Santos et al., 2017). It is obvious that much more time was devoted to defence during the preparations for World Cups, which only last a few weeks, because it is much easier to train and adopt defensive positions than it is for players to take attacking movements (Vergonis et al., 2019).

An efficient defence play, in order to decrease the opponents’ free playing area, can be accomplished by reducing the distance between the vertical lines and narrowing the space in width (Moura et al., 2012; Sarmento et al., 2018; Lepschy et al., 2021). This tendency towards compactness of the defence players is exactly what leads to an increase in the space behind the defence line and results in the open positions on the lateral sides. According to the data from the final pass analysis, the majority of goals from collective attacking actions (46.6%) were scored following deep passes, followed by crosses (39.1%), and back passes (14.3%). These findings are with line with previous studies (Durlik and Bieniek, 2014; Gonzalez-Rodenas et al., 2019) that’s showed that deep passes were the most frequent action performed by penultimate player in collective attacks.

The data relating goal scoring after individual actions have indicated that, regarding the individual characteristics of football players, the anticipation of events on the field, as is collecting a free ball, is of utmost importance for goal scoring in World Cups. Results of study which analyse goal scoring patterns in European elite soccer (Gonzalez-Rodenas et al., 2019) shown that most of the goals scored from individual actions comes from collecting a free ball, support our findings. Players who can perform quality shots from a distance can also demonstrate a special scoring quality. Dribbling is a technical and tactical element that shows the beauty of the football game and the players who are capable of dribbling through, i.e., outwitting their opponents are found to be very attractive for spectators. However, data show that this individual attribute has little bearing on goal scoring at World Cups. This is in line with findings of study. Gonzalez-Rodenas et al. (2019) where they found that goals scored after dribbling produced the 7.6% of goals. This fact may be due to the tactical development and greater defensive preparation in the current professional soccer, where disrupting the opponent to achieve shooting possibilities by means of individual actions require excellent skills (Barreira et al., 2014; Gonzalez-Rodenas et al., 2019).

This study has a multiple limitations. In the first place, the fact of using observational methodology may not capture the entire complexity of soccer actions and interactions, as previous studies based on ecological models have claimed (Glazier, 2010; Vilar et al., 2012). Also, specific limitation of our study is very specific selection of variables. In order to get a more complete picture of the development of football over a longer period of time, future studies need to be focused on a more comprehensive analysis of spatial and temporal as well as technical and tactical parameters which relate to starting and organising attacks leading up to a goal. The findings of such studies will contribute to a better understanding of the football evolution through history. In addition to that we would like to emphasise unequal total number of matches (there were more matches in recent competitions than in older ones) through the history of the World Cups which could bias the comparison.

## Conclusion

The results of the study have shown consistency for the World Cups played between 1966 and 2018 with regards to the following variables: *Game period* which is crucial for changing the score of a match (from the 76th minute until the end of a match), *Goal scoring zones* (most goals scored from inside the penalty area), and *Actions leading up to goals* (most goals scored by one touch finishing). Also, players’ individual characteristics which are essential to goal scoring at World Cups have been determined to be constant throughout football history, and they include: *Retrieving possession of a free ball* and *Shot from distance*. On the other hand, the findings of this study revealed that changes in physical, tactical, and technical terms over time caused a change in technical and tactical rules applied to the execution of goal-scoring attacking actions related to the *Type of play* and *Penultimate action*. An upward trend regarding the number of goals scored from set play and from collective attacking actions can be observed through the analysis of the last 14 FIFA World Cups. The rising quality of the team’s defence, which primarily reduces the opponent’s free playing area, can explain such an increase in goal scoring.

Based on the findings of this study, several recommendations can be made. First, coaches should ensure an adequate level of players’ physical readiness, which will enable them to endure the entire period of 90+ minutes of the match. Well-prepared players will be more focused and, as a result, make fewer mistakes. This especially refers to the period from the 76th minute until the end of the match. In terms of technical and tactical preparation for World Cups, coaches should focus on factors that can contribute to success whilst also being applied quickly from training to competition due to the limited time for national teams to practise. 11 versus 11 drills on a smaller pitch will enable a faster adjustment to offensive and defensive phases of the game. This kind of training requires quick decision making, prompt marking of the player in possession of the ball, diverse movement, and accurate passing during attacks. All of these factors contribute to the effectiveness of the collaborating open play, which is the most effective goal scoring strategy. Simultaneously, defensive play is being practised in response to the attacking phase of the game. The result of this study shown consistency in terms from which area in how goals were scored, accordingly to this the majority of the time spent training for individual improvement in finishing should be devoted to one touch finishing from the penalty box.

## Data availability statement

The raw data supporting the conclusions of this article will be made available by the authors, without undue reservation.

## Author contributions

All authors listed have made a substantial, direct, and intellectual contribution to the work and approved it for publication.

## Conflict of interest

The authors declare that the research was conducted in the absence of any commercial or financial relationships that could be construed as a potential conflict of interest.

## Publisher’s note

All claims expressed in this article are solely those of the authors and do not necessarily represent those of their affiliated organizations, or those of the publisher, the editors and the reviewers. Any product that may be evaluated in this article, or claim that may be made by its manufacturer, is not guaranteed or endorsed by the publisher.
