# Erratum: The Qatar 2022 World Cup warm-up: Football goal-scoring evolution in the last 14 FIFA World Cups (1966–2018)

**DOI:** 10.3389/fpsyg.2023.1156698

**Published:** 2023-02-10

**Authors:** Frontiers Production Office

**Affiliations:** Frontiers Media SA, Lausanne, Switzerland

**Keywords:** football (soccer), goal scoring, World Cup, tactics, performance analysis

Due to a production error, the DOI for this article was incorrectly registered as 10.3389/fpsyg.2023.954876. The correct DOI for the article is 10.3389/fpsyg.2022.954876.

The publisher apologizes for this mistake. The original version of this article has been updated.

